# En bloc Right Hemicolectomy with Pancreaticoduodenectomy for Advanced Ascending Colon Cancer

**DOI:** 10.70352/scrj.cr.24-0146

**Published:** 2025-04-05

**Authors:** Hiroyuki Takeda, Tetsuo Ishizaki, Ryutaro Udo, Tomoya Tago, Kenta Kasahara, Junichi Mazaki, Keiichiro Inoue, Yuichi Nagakawa

**Affiliations:** 1 Department of Gastrointestinal and Pediatric Surgery, Tokyo Medical University, Tokyo, Japan; 2 Inoue Geka-Naika Clinic, Tokyo, Japan

**Keywords:** colon cancer, right hemicolectomy, pancreaticoduodenectomy, en bloc resection

## Abstract

**INTRODUCTION:**

While simultaneous complex surgical procedures such as right hemicolectomies with pancreaticoduodenectomies (RHPD) may increase overall surgical complexity, complications, and risk of death, it is the only cure for advanced ascending colon cancer (AACC) that has directly invaded the duodenum/pancreas. There are a few reports, especially from Japan. Here, we report an extremely rare case of a patient who underwent RHPD for AACC with direct invasion to the duodenum and liver and describe the patient’s long-term survival after en bloc resection.

**CASE PRESENTATION:**

The patient was a 76-year-old man who presented with a chief complaint of right abdominal pain and weight loss of 12 kg over the past month. Colonoscopy revealed the entire circumference of a type 2 tumor in the ascending colon. Preoperative computed tomography showed a 12 cm mass lesion with wall thickening in the ascending colon which was also invading the second portion of the duodenum. MSI-H/dMMR was negative. RHPD and partial hepatectomy were performed with open surgery because of a preoperative diagnosis of clinical T4b (duodenum and liver) N1bM0 stage IIIc cancer. Although grade 2 adverse effects, which delayed gastric emptying was observed during the patient’s postoperative course, the patient’s condition resolved through conservative therapy. Oral intake started on postoperative day 17, and the patient was discharged on postoperative day 25. Capecitabine plus oxaliplatin was administered as adjuvant chemotherapy for 6 months. Hematoxylin and eosin staining revealed moderately differentiated adenocarcinoma invading the duodenum and liver. The patient was diagnosed as pathological T4b (duodenum and liver) N1bM0 stage IIIc cancer. No recurrence was noted up to 40 months after the surgery.

**CONCLUSIONS:**

The only curative therapy for AACC with involvement of the duodenum is en bloc RHPD. Here, we described a case in which long-term survival was achieved by ensuring R0 with en bloc resection.

## Abbreviations

AACC
: advanced ascending colon cancer
CT
: computed tomography
PD
: pancreaticoduodenectomy
RHPD
: right hemicolectomy with pancreaticoduodenectomy

## INTRODUCTION

Colorectal cancer is a highly prevalent disease worldwide.^1)^ Among the cases, 0.18%–0.28% have been reported to show direct invasion into the duodenum^2,3)^ and 0.04% have been found to show involvement of the liver.^2)^ These adhesions may be both frank tumor infiltration and due to peritumoral inflammation. In terms of histologic findings, tumor infiltration into adjacent organs has been reported in only 53.4%–63.6% of patients who underwent multivisceral resection for locally advanced colon cancer, while the remaining patients were reported to have only adhesions due to local inflammations.^4,5)^ On the contrary, it is worth mentioning that a study found 95% true tumoral invasion into the duodenum/pancreas in patients who underwent pancreaticoduodenectomy (PD) and concurrent colectomies for colon cancer.^6)^ There is no way to accurately distinguish these findings intraoperatively. Therefore, the standard management approach is en bloc resection of the diseased organ along with that of the adjacent organ with infiltration.^7)^ R0 resection could result in a favorable prognosis.^2,4,8)^ Right hemicolectomy with PD (RHPD) for advanced ascending colon cancer (AACC) with direct invasion to the duodenum/pancreas have been performed in 0.3%–2.6% of patients treated with curative intent with positive results.^8)^ There are a few reports, especially from Japan. Here, we report an extremely rare case of a patient who underwent RHPD for AACC with direct invasion into the duodenum and liver and describe the patient’s long-term survival after en bloc resection.

## CASE PRESENTATION

**Fig. 1:**
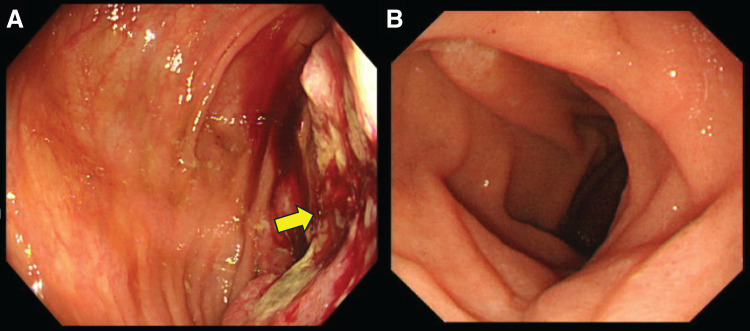
(**A**) Colonoscopy revealed the entire circumference of a type 2 tumor in the ascending colon (arrow). (**B**) Esophagogastroduodenoscopy showed no tumor infiltration in the duodenum.

**Fig. 2:**
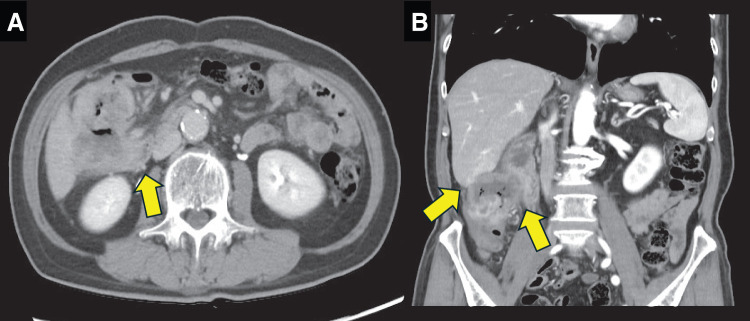
(**A**) Axial and (**B**) coronal computed tomography showed a 12-cm mass lesion with wall thickening in the ascending colon invading the second portion of the duodenum, and a small extent of invasion into the liver was also observed (arrows).

**Fig. 3:**
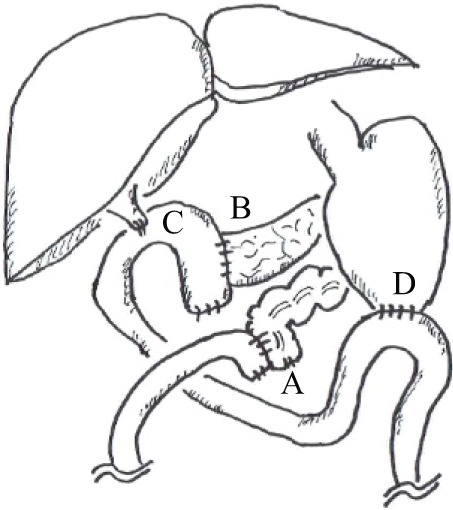
Right hemicolectomies with pancreaticoduodenectomies, partial hepatectomy, and D3 lymph node dissection were performed. Reconstruction was shown. (**A**) Functional end-to-end anastomosis. (**B, C, D**) Modified Child’s method.

**Fig. 4:**
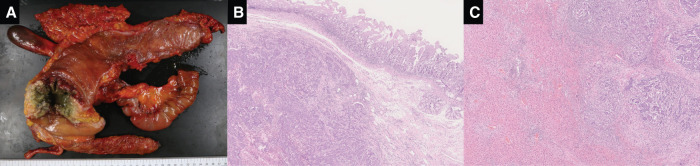
Resected specimen and histopathological findings. (**A**) Macroscopic finding showed a type 2 tumor in the ascending colon measuring 12 cm. (**B, C**) Hematoxylin and eosin staining revealed moderately differentiated adenocarcinoma invading the duodenum and the liver.

The patient was a 76-year-old man who presented with a chief complaint of right abdominal pain and weight loss of 12 kg over the past month. His medical history included diabetes and Parkinson’s disease, and his American Society of Anesthesiologists physical status was class 2. Levels of the tumor markers cancer embryonic antigen and carbohydrate antigen were within the normal range. Colonoscopy revealed the entire circumference of a type 2 tumor in the ascending colon (**Fig. 1A**). Histological examination revealed a moderately differentiated adenocarcinoma. This patient was of non-MSI-H/dMMR status. Esophagogastroduodenoscopy showed no tumor infiltration in the duodenum (**Fig. 1B**). Computed tomography (CT) showed a 12 cm mass lesion with wall thickening in the ascending colon invading the second portion of the duodenum (**Fig. 2A**), and a small extent of invasion into the liver was also observed (**Fig. 2B**). The decision to perform the surgery was made after a thorough risk assessment in conference with the colorectal surgeon, the hepato-biliary-pancreatic surgeon, and the anesthesiologist. The decision for upfront surgery was made in conference with the colorectal surgeon, the hepato-biliary-pancreatic surgeon, and the radiologist because R0 resection was possible on preoperative imaging studies. Open surgery was performed because of a preoperative diagnosis of clinical T4b (duodenum and liver) N1bM0 stage IIIc cancer according to the guidelines of the Japanese Society for Cancer of the Colon and Rectum.^9)^ During the surgery, it was found that the AACC was extensively invading the second portion of the duodenum. Because there was no noncurative factor, RHPD, partial hepatectomy, and D3 lymph node dissection for AACC were performed. The duration of the surgery was 451 min, and the amount of blood loss was 949 mL. Reconstruction was performed through functional end-to-end anastomosis following the modified Child’s technique (**Fig. 3**). Macroscopic findings showed a type 2 tumor in the ascending colon measuring 12 cm (**Fig. 4A**). Hematoxylin and eosin staining revealed moderately differentiated adenocarcinoma invading the duodenum (**Fig. 4B**) and liver (**Fig. 4C**). The patient was diagnosed as pathological T4b (duodenum and liver) N1bM0 stage IIIc cancer. Although grade 2 adverse effects, which delayed gastric emptying was observed during the patient’s postoperative course, the patient’s condition resolved through conservative therapy. Oral intake started on postoperative day 17, and the patient was discharged on postoperative day 25. Capecitabine plus oxaliplatin was administered as adjuvant chemotherapy for 6 months. No recurrence was noted up to 40 months after the surgery.

## DISCUSSION

In this report, we described long-term survival after en bloc resection in the rare case of a patient who underwent RHPD for AACC involving the duodenum and the liver. The first case in which RHPD was performed for AACC was reported in 1953.^10)^ A summary of the previous literatures in RHPD for AACC is shown^11–14)^ in **Table 1**. It indicated high negative resection margin rates (94.4%–100%) and the 5-year overall survival rate of patients who underwent RHPD for AACC was 52.6%–58.0%. The results are comparable to those in cases of colon cancer without the involvement of other organs.^15,16)^ In a few systematic reviews, several patients with AACC who underwent colectomy, with associated PD, had high negative resection margin rates (95.5%–97.5%) and a median overall survival period of 70–168 months.^6,17)^ Meanwhile, the prognosis was poor for patients in whom complete resection could not be achieved, with their median survival being 11.6–13.9 months.^18,19)^ Therefore, even if AACC involves invasion of other organs, RHPD is meaningful if R0 and en bloc resection is possible.

**Table 1:** Postoperative outcomes of previous reports

Authors	Number	Operation time (minutes)	Operative blood loss (mL)	Complete resection rate (%)	OC (%)	PF (%)	DGE (%)	Ileus (%)	POHS (days)	5OS
Lee^11)^	9	320 (200–420)	700 (100–2000)	9 (100)	N/A	N/A	N/A	2 (22.2)	N/A	56.0%**
Sheng^12)^	7	410 ± 64	514 ± 157	N/A	5 (71.4)	N/A	N/A	N/A	22.1 ± 7.2	N/A
Yan^13)^	19	320 (222–410)	268 (100–600)	19 (100)	14 (73.7)	9 (47.4)	N/A	1 (5.2)	23.5 (11–45)	58.0%
Chen^14)^	19*	450 (372–708)	420 (150–3130)	18 (94.4)	8 (42.1)	2 (10.5)	3 (15.8)	N/A	19 (9–45)	52.6%

*non-emergency group, **3OS: 3 years overall survival rate, Described as median and range or mean and standard deviation.

5OS, 5 years overall survival rate; DGE, delayed gastric empty; OC, overall complications; PF, pancreatic fistula; POHS, post operative hospital stay

In several patients with AACC and limited duodenum invasion (apart from the ampulla of Vater proximity), a partial duodenectomy can be an alternative to PD to obtain negative resection margins. A systematic review published in 2014 comparing right colectomy with PD versus partial duodenectomy for AACC showed similar results in terms of postoperative complications for both groups of patients.^20)^ However, the long-term prognosis appeared less favorable for the group of patients who underwent partial duodenectomy, albeit the difference was not statistically significant. Similar long-term outcomes were recently confirmed by a review published in 2023.^21)^ Colon cancer is classified into 3 categories according to the degree of duodenal involvement:^22)^ type I, involvement of less than half of the circumference of the lateral duodenal wall; type II, involvement of more than half of the circumference away from the papilla; and type III, involvement of more than half of the circumference close to the papilla. Type I is managed with sleeve resection, type II with segmental resection, and type III with PD. Partial duodenectomy is recommended for types I and II. In this case, we found extensive invasion of the duodenum on preoperative CT, and we chose RHPD for the diagnosis of type III.

The advantages of preoperative therapy are as follows: shrinking tumors before surgery may reduce the risk of incomplete resection, effectiveness in eradicating micrometastases and response to preoperative therapy, unlike adjuvant therapy, is observable, so could potentially guide subsequent treatment decisions.^23,24)^ However, there is no clear evidence for colon cancer in preoperative therapy like rectal cancer.^25,26)^ We noted the following disadvantages of preoperative therapy. Progression in disease reduces the chance of surgical cure, and toxicity during neoadjuvant therapy compromises fitness for surgery, increasing perioperative complications. In this case, the decision for upfront surgery was made in conference with the colorectal surgeon, the hepato-biliary-pancreatic surgeon, and the radiologist because the evidence for neoadjuvant therapy for T4b colon cancer was not established and R0 resection was possible on preoperative imaging studies. This patient was non-MSI-H/dMMR, however, if positive, the effectiveness of the neoadjuvant immune checkpoint inhibition for T4b colon cancer with mismatch repair deficiency has been reported in terms of disease-free survival and relatively longer overall survival.^27)^ This may become a therapeutic strategy of interest for T4b colon cancer in MSI-H in the future.

Although rarely performed together, simultaneously performing complex surgical procedures such as RHPD may increase the operation’s overall complexity, complications, and risk of mortality. The factors associated with improved overall survival after PD and associated colectomies for locally advanced right colon cancer were well- and moderately differentiated tumors, the absence of lymph node metastases, and adjuvant chemotherapy. Histological proof of pancreatic invasion and severe postoperative complications adversely influence long-term outcomes after PD with concurrent colectomies for colon cancer.^22)^ A few systematic reviews have shown overall complications, pancreatic fistula, delayed gastric emptying, and ileus rates of 42.1%–73.7%, 10.5%–47.4%, 15.8%, and 5.2%–22.2%, respectively, after RHPD for AACC.^11–14)^ Postoperative complications may worsen patients’ early outcomes, reduce quality of life after RHPD, and adversely affect long-term oncological outcomes. Thus, severe postoperative complications may decrease adjuvant chemotherapy rates in patients who have undergone RHPD, leading to increased recurrence rates and decreased survival.^28,29)^ Carefully selecting patients suitable for RHPD is important to reduce the potentially severe complications of surgical procedures and maximize oncological benefits.^22)^ In this case, accurate preoperative imaging and adequate reserve of each organ were ensured before surgery. The high-quality surgical skills and stable postoperative outcomes associated with our experienced pancreatic surgery team, which performs more than 150 pancreatic surgeries for pancreatic tumors per year in our hospital, were also factors in our decision to perform RHPD in this case. It is important to minimize the occurrence of postoperative complications and, if they do occur, to resolve them with early intervention and early introduction of adjuvant chemotherapy. Thus, coordination between the colorectal surgeon and the hepato-biliary-pancreatic surgeon in a high-volume center is an important factor for the safe and feasible completion of this complex surgical procedure.

## CONCLUSIONS

The only curative therapy for AACC with involvement of the duodenum is en bloc RHPD. Here, we described a case in which long-term survival was achieved by ensuring R0 with en bloc resection. To reduce the potentially severe complications of surgical procedures and maximize oncological benefits, collaborative effort between a colorectal surgeon and a hepato-biliary-pancreatic surgeon is critical.

### ACKNOWLEDGMENTS

The authors thank Editage (https://www.editage.jp) for English language editing.

## DECLARATIONS

### Funding

The authors received no specific funding for this work.

### Authors’ contributions

Conception and design: T.I.

Data acquisition: J.M.

Data interpretation: R.U., T.T., and K.K.

Drafting the manuscript: H.T.

Supervision of the manuscript: K.I. and Y.N.

All authors have read and approved the manuscript, and they are responsible for the manuscript.

### Availability of data and materials

The data will be made available on reasonable request.

### Ethics approval and consent to participate

This report has been performed in accordance with the Declaration of Helsinki, and was approved by the Ethical Committee of Tokyo Medical University (Approval No.: T20-0054).

### Consent for publication

Informed consent to publish has been obtained.

### Competing interests

The authors declare that they have no competing interests.
